# Poststroke Seizure and Epilepsy: A Review of Incidence, Risk Factors, Diagnosis, Pathophysiology, and Pharmacological Therapies

**DOI:** 10.1155/2022/7692215

**Published:** 2022-10-26

**Authors:** Joseph Phan, Mario Ramos, Theodore Soares, Mayur S. Parmar

**Affiliations:** Dr. Kiran C. Patel College of Osteopathic Medicine, Nova Southeastern University, Tampa Bay Regional Campus, Clearwater, Florida, USA

## Abstract

Stroke is the most common cause of epilepsy and ultimately leads to a decrease in the quality of life of those affected. Ischemic and hemorrhagic strokes can both lead to poststroke epilepsy (PSE). Significant risk factors for PSE include age < 65age less than 65 years, stroke severity measured by the National Institutes of Health Stroke Scale (NIHSS), cortical involvement, and genetic factors such as TRPM6 polymorphism. The diagnosis of PSE is made by using imaging modalities, blood biomarkers, and prognostic criteria. Electroencephalography (EEG) is currently the gold standard to diagnose PSE, while new combinations of modalities are being tested to increase diagnostic specificity. This literature review uncovers a newly found mechanism for the pathology of poststroke epilepsy. The pathogenesis of early-onset and late-onset is characterized by sequelae of neuronal cellular hypoxia and disruption of the blood-brain barrier, respectively. Interleukin-6 is responsible for increasing the activity of glial cells, causing gliosis and hyperexcitability of neurons. Epinephrine, high-mobility group protein B1, downregulation of CD32, and upregulation of HLA-DR impact the pathology of poststroke epilepsy by inhibiting the normal neuronal immune response. Decreased levels of neuropeptide Y, a neurotransmitter, act through multiple unique mechanisms, such as inhibiting intracellular Ca^2+^ accumulation and acting as an anti-inflammatory, also implemented in the worsening progression of poststroke epilepsy. Additionally, CA1 hippocampal resonant neurons that increase theta oscillation are associated with poststroke epilepsy. Hypertensive small vessel disease may also have an implication in the temporal lobe epilepsy by causing occult microinfarctions. Furthermore, this review highlights the potential use of statins as primary prophylaxis against PSE, with multiple studies demonstrating a reduction in incidence using statins alone, statins in combination with antiepileptic drugs (AEDs), and statins with aspirin. The evidence strongly suggests that the second generation AEDs are a superior treatment method for PSE. Data from numerous studies demonstrate their relative lack of significant drug interactions, increased tolerability, and potential superiority in maintaining seizure-free status.

## 1. Introduction

Strokes are the most common etiology of epilepsy in patients 65 and older. In rare situations, ischemic or hemorrhagic strokes result in complications of epilepsy, which is known as poststroke epilepsy (PSE). According to the most widely accepted diagnostic criteria, PSE is diagnosed by the occurrence of two or more seizures with a greater than 60% risk of a subsequent seizure occurring in the next decade after a cerebrovascular accident. Early-onset seizures, defined as occurring less than one week following a stroke, carry a 33% risk of additional seizures in the next ten years and therefore, do not qualify as PSE. However, they are considered a significant risk factor for developing PSE. Its pathophysiology stems from metabolic and electrolyte derangement associated with acute infarction [1, 2]. On the other hand, late-onset seizures, defined as occurring more than one week following a stroke, carry a 71.5% risk of additional seizures occurring in the next ten years, thereby falling into the criteria to diagnose PSE [3]. Unlike early-onset seizures, late-onset seizures originate from areas of the injured brain where networks have undergone structural alteration, such as the disruption of the blood-brain barrier (BBB) [1, 4].

The purpose of distinguishing early- and late-onset seizures is to categorize their different risk factors and pathophysiology so that treatment can be tailored appropriately. To simplify the recent research about poststroke epilepsy, our literature review has conglomerated multiple clinical and observational studies to provide a comprehensive overview so that further research can be conducted easily.

## 2. Review Outline

### 2.1. Risk Factors, Etiology, and Sequelae of Poststroke Epilepsy

The time period with the highest risk for the development of epilepsy after a stroke is during the first 2 years. Stroke is the most common etiology of epilepsy in patients over the age of 65 years old [5]. The prevalence of poststroke seizures increases with various factors such as younger age (<65 years), male gender, and cortical involvement, primarily parietal lobe involvement. No significant difference has been observed in geographic regions and patient race and ethnicity. In adult populations, seizures were more prevalent in hemorrhagic stroke than ischemic stroke. Genetic factors that increase the risk for poststroke seizures (PSS) are overexpression of aldehyde dehydrogenase 2 and the presence of TRPM6 polymorphism [6]. A high National Institutes of Health Stroke Scale (NIHSS) score and alcoholism have been correlated to an increased risk of early seizures. In a total of 1066 patients with a mean age at index stroke of 55.8 and a mean National Institutes of Health Stroke Scale (NIHSS) score of 5.3, at 8-year follow-up, 7.9% of the patients had developed poststroke epilepsy with 0.6% developing status epilepticus [5]. Moderate and severe disability based on deficits in visual, communication, motor, and/or sensory domains was found to be predictive of poststroke seizures based on a cohort study involving 469 stroke patients conducted from 1982 to 2003 with 20 years of follow-up analyzed the incidence of seizures following stroke.

#### 2.1.1. Risk Factors and Etiologies

*(1) Type of Strokes*. Hemorrhagic strokes occur when a blood vessel that supplies the brain ruptures and bleeds, and brain cells and tissues do not receive nutrients and oxygen [7]. On the other hand, ischemic strokes are caused by a blockage of an artery or vein and impair blood flow to the brain [8]. Focal aware seizures were defined as seizures not leading to a loss of consciousness, while focal impaired-awareness seizures involved a change or loss of consciousness or awareness. Generalized tonic-clonic seizures occurred in 65% of patients with ischemic stroke. However, focal impaired-awareness seizures were the most common (40%) among patients with hemorrhagic strokes [9]. Early-onset seizures were significantly associated with atrial fibrillation (AF), as patients with AF showed a statistically higher score on the modified Rankin scale, a predictive score for the risk of epileptic seizures. Patients with early-onset seizures had a statistically higher score on the modified Rankin scale than patients with late-onset seizures. There was no significant correlation between the type of epileptic seizure (generalized, focal aware, and focal impaired awareness) and the score on the modified Rankin scale [10].

*(2) Age and Sex*. Age greater than 65 years is a protective factor for seizure recurrence due to a smaller cerebral cortex and decreased excitability induced by degenerative changes in elderly individuals (Table 1). 71% of patients who developed acute symptomatic or unprovoked seizures after ischemic strokes were 65 years or younger. A reason for this may be that younger patients (<65 years) are more likely to experience large cortical infarcts rather than small vessel disease and that this may contribute to subsequent epilepsy development and a higher risk of seizure recurrence [11]. However, younger patients have a higher risk for PSE due to ischemic stroke secondary to artery dissection [5]. Female sex may impact predisposition to certain types of arterial ischemic stroke in patients. The odds of poststroke seizures in females with total anterior circulation infarct (TACI) and posterior circulation infarct (POCI) were eight-fold higher than in females with lacunar anterior circulation infarct (LACI) and partial anterior circulation infarct (PACI) stroke (odds ratio (OR): 8.62). Girls with TACI were also more likely to develop seizures than boys with TACI (56% vs. 28%) [12, 13].

*(3) Cortical and Vascular Risk Factors*. Posthemorrhagic stroke seizures were more likely to develop in patients with larger lesions involving multiple lobes of the brain than in those with single lobar involvement (Table 1). Patients were also more likely to have a cortical lesion in the frontal lobe, but this is associated with hemorrhagic strokes rather than ischemic strokes [14]. The location of the infarct within the cerebral vasculature does not play a significant role in developing PSE. However, in certain cases, it is found that the middle cerebral artery infarct is a risk factor for poststroke seizures [11]. Additionally, total anterior circulation infarction (TACI), partial anterior circulation infarction (PACI), and carotid artery dissection were independently associated with PSE [5].

Cardiovascular risk factors such as a history of myocardial infarction (MI), hypertension (HTN), and left ventricular hypertrophy (LVH) were associated with late-onset seizures. Conditions such as transient ischemic attack (TIA) and intracranial venous thrombosis (IVT) were not associated with the occurrence of poststroke seizures. Coronary heart disease (CHD) has been more commonly associated with PSE. CHD generation is due to a casual association with a vascular lesion caused by atrial fibrillation or heart failure [5, 15]. With these above considerations in mind, physicians should factor these risks in patient care as these pose additional risks to the patient, such as further neuronal damage and neurological deterioration.

Hypertension is a known risk factor for developing PSE. Males have an increased risk of developing PSE due to their higher risk of head injuries, stroke, and CNS infections (Table 1). A blood pressure cutoff of greater than 130/80 mmHg was associated with a 126% higher risk of developing PSE. The highest risk was associated with a systolic blood pressure reading between 160 and 179 mmHg. Thus, antihypertensive medications such as angiotensin-converting enzyme (ACE) inhibitors, angiotensin receptor blockers (ARBs), and calcium channel blockers (CCB) have a protective association, and there may be a beneficial effect of obtaining optimal blood pressure control after a stroke. Further studies are needed to corroborate these novel associations. [15, 16]. Hyperglycemia upon admission for cerebrovascular infarction can also predict PSE due to aggravating damage to neuronal cells because of oxidative stress, increased blood-brain barrier permeability, and inflammatory response [17]. Rat models demonstrated that hyperglycemia could have a three to five-fold increase of tissue plasminogen activator- (tPA-) induced brain hemorrhage in postischemic brains, thus providing a rationale for glucose control in hyperglycemic stroke patients.

*(4) Genetic Involvement of Aldehyde Dehydrogenase 2 and TRPM6 Polymorphism*. A family history of seizures strongly correlates with PSE, suggesting some degree of genetic contribution. About 30% of all epilepsies are believed to be of genetic origin. One such example is the single nucleotide polymorphism rs671 of aldehyde dehydrogenase 2 (ALDH2). The association of PSE and ALDH2 rs671 polymorphism is due to its relationship with elevated levels of 4-hydroxy-2nonenal (4-HNE), which is a specific marker of oxidative stress generated by lipid peroxidation. The high levels of 4-HNE in PSE patients decrease ALDH2 activity by thiol oxidation. The decreased activity of ALDH2 then increases reactive oxidative species, malondialdehyde, and apoptosis causing ischemic brain damage and the development of poststroke epilepsy. This suggests that ALDH2 rs671 polymorphism and plasma 4-HNE can be used as predictive markers for PSE. Additionally, pharmaceuticals that activate ALDH2 may be a potential therapy to prevent PSE [18, 19].

Furthermore, the polymorphism of TRPM6 (transient receptor potential cation channel subfamily M member 6) rs2274924 increases the susceptibility of PSE (Table 1). Sequencing of the TRPM6 identified 3 genotypes: TT, CT, and CC where the frequency of CC genotype and C alleles in patients with PSE were significantly higher. The possible mechanism for the association of polymorphism TRPM6 rs2274924 C allele and PSE is TRPM6's role as an ion channel subunit regulating magnesium (Mg^2+^) and calcium (Ca^2+^)in the intestinal tract and kidneys. The TRPM6 rs2274924 C allele may alter the protein conformation and reduce its ion channel activity. Additionally, the rs2274924 C allele polymorphism of TRPM6 is associated with lower levels of Mg^2+^ which can decrease the threshold for seizure activity. Therefore, low serum Mg^2+^ in rs2274924 C allele polymorphism of TRPM6 may be a predictor of PSE. Future studies should verify if oral Mg^2+^ supplementation in these patient populations may effectively prevent PSE [6, 20].

*(5) Reperfusion Therapy*. The risk of PSE increases with ineffective systemic thrombolysis, resulting in large and diffuse cerebral infarcts with hemorrhagic components [21]. In patients with PSE who had undergone systemic thrombosis, aphasia was the most often diagnosed symptom due to the infarct being more often located in the left hemisphere in patients with PSE. Moreover, in patients with PSE receiving systemic thrombolysis, there seems to be a hemorrhagic component in the deep and terminal branches of the middle cerebral artery, which has been attributed to causing greater permeability of the blood-brain barrier in PSE. In patients who have undergone systemic thrombolysis, the hemorrhagic component may have contributed to the PSE by acting as a neurotoxin in the cerebral cortex, activating matrix metalloproteinases, losing inhibitory neurons, and increasing the synthesis of nitric oxide [21, 22].

Systemic thrombolysis in patients with PSE was more likely to develop generalized and bilateral tonic-clonic seizures with alteration in consciousness. In contrast, patients who received medical therapy with antiepileptic drugs (AED) were more likely to develop focal seizures without alteration in consciousness. Surprisingly, due to its increased risk of bilateral clonic-tonic seizures, the systemic thrombolysis group usually has an early start on antiepileptic therapy with proper therapeutic doses and high patient adherence, in stark contrast to the nonthrombolysis group [22].

Hemorrhagic transformation is a complication in patients with ischemic stroke that occurs when blood extravasates across the blood-brain barrier into the brain. Poststroke seizures were independently associated with hemorrhagic transformations and higher stroke severity, as defined by a modified Rankin Scale (mRS) greater than 2 at 3 months poststroke. Patients who experienced hemorrhagic transformation after thrombolysis have an increased risk for seizures and poststroke epilepsies (odds ratio (OR): 3.26). Thrombolytic therapy is associated with an increased chance of hemorrhagic transformation in ischemic stroke, while bleeding is associated with an increased risk of PSE [23]. Although past studies have shown a correlation between reperfusion therapy and poststroke epilepsy, this may be due to treatment selection bias, as perfusion therapy is more likely to be performed in those with more severe cerebral infarcts. After correcting for reperfusion therapy propensity, there was no difference in acute or poststroke epilepsy risk. This also did not reduce the risk of PSE because, despite removing the thrombus, there may be ongoing excitotoxicity, inflammation, or blood-brain disruption that will lead to the development of an epileptogenic network [14].

*(6) COVID-19*. Post-COVID-19 acute ischemic and hemorrhagic strokes may be associated with seizure onset. In a case study, only 5 patients with an ischemic and hemorrhagic stroke presented with seizure onset. This may be due to hypoxemia and excessive secretion of inflammatory markers during COVID-19, which contributes to the onset of acute stroke and increases the incidence of seizures [24].

*(7) Early Seizure Determines the Risk of PSE and Sequelae of Poststroke Status Epilepticus*. Poststroke status epilepticus (PSSE) is a seizure that lasts more than 5 minutes or has more than 1 seizure within a 5-minute period without returning to a normal level of consciousness. PSSE duration greater than 16 hours are more likely to develop seizures in patients with early-onset status epilepticus. Nearly a quarter of patients with first-time early-onset PSSE developed PSE [25]. A higher risk of developing PSE was independently associated with an NIHSS score greater than 4 at stroke presentation, as the estimated seizure relapse rate within the first year of follow-up was higher in these patients [26]. More studies are needed to conclude if the likelihood of recurrent seizures is higher after early-onset PSE than after a single early-onset poststroke seizure.

*(8) Mortality due to PSE*. PSE is an independent predictor of mortality. Patients with PSE were more likely to die of a pneumonia-related cause than those without PSE (56% vs. 37%), but mortality due to cardiovascular events excluding ICH sequelae was not increased (42% vs. 39%). Other risk factors for increased mortality were higher age at ICH onset, male sex, the poorer functional outcome at 3 months, no subcortical ICH, diabetes, and cancer prior to ICH [27].

### 2.2. Diagnosis of PSE

The diagnosis of PSE is typically made by a neurologist or epileptologist who receives referrals of patients with new-onset seizures from emergency departments or general practitioners. A diagnosis of early-onset poststroke seizures is made if the seizure occurs within two weeks and are provoked seizures. In contrast, late-onset poststroke seizures occur after two weeks and are unprovoked seizures. The International League Against Epilepsy stated that the diagnosis requires a risk of seizure recurrence exceeding 60%. They revised their old definition of epilepsy in 2014. They added that a diagnosis could be made with one unprovoked seizure and a probability of further seizures similar to the general recurrence risk (at least 60%) after two unprovoked seizures occurring over the next 10 years [28].

#### 2.2.1. Diagnosing Based on Electroencephalogram

Electroencephalogram (EEG) is an important diagnostic method for detecting seizures, and this tool is often used in combination with Computed Tomography (CT) and Magnetic Resonance Imaging (MRI) to detect abnormalities causing epileptic activity [3]. EEGs are also useful in assessing the risk of seizures in poststroke patients. EEG showing periodic lateralized and bilateral independent periodic lateralized epileptiform discharges were prone to a higher risk of developing poststroke seizures. A normal EEG does not exclude epileptogenicity. Patients with focal epileptiform EEG findings and experiencing partial-onset seizures have a greater seizure recurrence rate [29, 30]. Moreover, patients with anterior circulation ischemic strokes and early EEG background asymmetry within the first 72 hours have a 3.2 times higher risk of an unprovoked seizure. If the early EEG shows epileptiform discharge, there is a 3.8 times greater risk of seizures than patients with a normal early poststroke EEG [29].

It is critical to perform EEG monitoring for at least 24-48 hours poststroke in patients admitted to the intensive care unit (ICU) if there are suspected clinical paroxysmal events or unexplained altered mental status. Asymmetrical background and interictal epileptiform activity detected on EEG performed during the first 72 hours poststroke were independent predictors of PSE during the first year following the event [29]. In comatose patients, patients with periodic discharges, or patients who are pharmacologically sedated, a more prolonged EEG of over 48 hours may lead to the detection of nonconvulsive seizures.

#### 2.2.2. Role of Computed Tomography (CT) and Magnetic Resonance Imaging (MRI) in Diagnosis

In an acute setting, cranial CT is used to rapidly image patients with seizures to rule out intracranial hemorrhage (ICH) [31]. Perfusion CT can also help differentiate stroke, stroke mimics, and status epilepticus, but it must be performed at a strict interval of 3 hours before seizure onset to improve sensitivity. In conjunction with EEG and clinical findings, the computed tomography is a useful diagnostic tool to establish the cause of the first seizure in adults. MRI of the brain is the most sensitive noninvasive imaging modality of choice because it will show several abnormalities that may be missed on CT, such as cortical malformations, hippocampal sclerosis, small mass lesions, and cavernomas. Postictal single-photon emission computed tomography (SPECT) can potentially complement EEG in accurately diagnosing PSE, especially in patients without epileptiform discharges on EEG. SPECT also has a wide diagnostic window, allowing diagnosis even days after the seizure ends [31, 32].

The parietal lobe has a critical role in the epileptogenesis of PSE and is a significant independent predictor of PSE. MRI can be used to measure the dysfunction of the parietal lobe and its implication on the development of PSE [33]. The parietal lobe contains a prominent intraparietal tract connecting the postcentral gyrus to both the supramarginal and angular gyri of the inferior parietal lobule. These tracts extend into the ipsilateral insular cortex, nucleus reticularis thalami, and lateral posterior thalami exhibit high-intensity lesions on brain MRI of status epilepticus; thus, a dysfunction in the parietal lobe due to infarction may play a critical role in the epileptogenesis of PSE. The involvement of the parietal lobe in infarction was significantly higher in the PSE group than in the stroke without seizure (SWS) group. However, there are no significant differences in the infarction volume [17].

#### 2.2.3. SeLECT Score and Interleukin-1*β*

**Figure 1:**
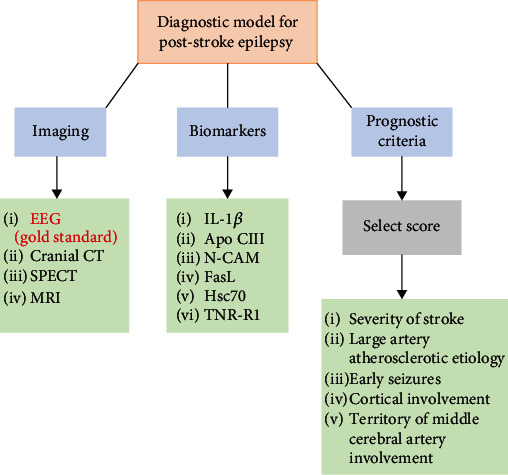
The main tools for diagnosing PSE: imaging, blood biomarkers, and prognostic models. The gold standard for diagnosing PSE is EEG, while CT is often used in an acute setting. Combining several of these modalities (e.g., IL-1*β* and SeLECT score) results in greater sensitivity and specificity for diagnosis. The SeLECT score ranges from 0-9, with 9 indicating the highest risk for developing PSE. High levels of IL-1*β*, APO CIII, NCAM, and FasL and low levels of Hsc70 and TNR-R1 are used to diagnose PSE. Abbreviations: EEG: electroencephalography; SPECT: single-photon emission computerized tomography; CT: computed tomography; MRI: magnetic resonance imaging; IL-1*β*: interleukin-1*β*; APO CIII: apolipoprotein CIII; NCAM: neural cell adhesion molecule; FasL: fas ligand; Hsc70: heat shock 70 kDa protein-8; TNR-R1: tumor necrosis factor receptor 1; and PSE: poststroke epilepsy.

Further studies are warranted to assess whether incorporating EEG findings with existing models, such as the SeLECT score, could improve predictive accuracy (Figure 1). The SeLECT score is a novel clinical tool for predicting late seizure incidence after an ischemic stroke. This score can be used to predict the risk of PSE by analyzing five criteria: severity of the stroke, large artery atherosclerotic etiology, presence of early seizures, cortical involvement, and territory of middle cerebral artery involvement. This score ranges from 0–9, with higher values indicating a higher risk of PSE [33]. IL-1*β* is a critical inflammatory cytokine during neuroinflammation after ischemic stroke and is associated with an increased risk of PSE. The combined SeLECT score and IL-1*β* prediction were significantly higher than the independent prediction of the SeLECT score or IL-1*β* [34].

#### 2.2.4. Blood Biomarker—Neural Cell Adhesion Molecules and Tumor

Patients with early-onset seizures had high levels of D-dimer, apolipoprotein CIII (Apo CIII), neural cell adhesion molecule (NCAM), and Fas ligand (FasL) and lower levels of heat shock 70 kDa protein-8 (Hsc70) and tumor necrosis factor receptor 1 (TNR-R1) (Figure 1). However, only high levels of NCAM and low levels of TNR-R1 were independently associated with developing early-onset seizures after an acute stroke event. NCAMs play a role in synaptic activity, and this observation may be due to an increased number of synapses in early seizures. Lower levels of TNF-R1 were also found in patients with early-onset seizures. This can be explained since most of these receptors would be bound to TNF-alpha during neuroinflammation associated with poststroke seizures. An adjusted logistic regression model was used to identify clinical variables, and this model's predictive power was greater when combined with these blood biomarkers than when used alone (73.5% vs. 64%) [35].

### 2.3. Poststroke Epileptogenesis

**Figure 2:**
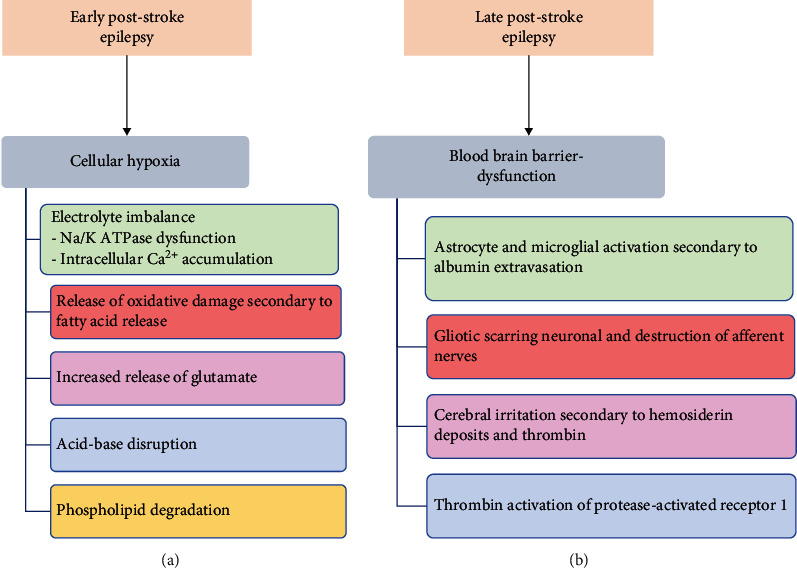
(a) General pathophysiology of early poststroke epilepsy (PSE). Early PSE is often due to cellular hypoxia secondary to reduced blood supply caused by a hemorrhagic or ischemic stroke. (b) General pathophysiology of late PSE. Late PSE is often a complication of blood-brain barrier dysfunction resulting in multiple glial cell dysfunction (as listed above).

**Figure 3:**
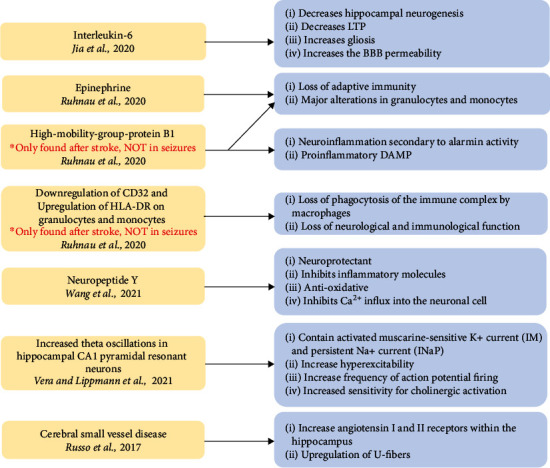
Current discovered pathological mechanisms of poststroke epilepsy gathered from 2017-2021. This figure reveals each pathological factor: interleukin-6 (IL-6), epinephrine, high-mobility group protein B1 (HMGB-1), downregulation of CD32, upregulation of HLA-DR on granulocytes and monocytes, neuropeptide Y, increased theta oscillation in hippocampal CA1 pyramidal resonant neurons, cerebral small vessel disease, and the mechanism by which they disrupt normal neurological function. Each new pathological discovery reveals a dysfunction of the immune system, causing gliosis, neuroinflammation, and loss of adaptive immunity. Note that HMGB-1 is not found after a seizure episode but after a recent stroke.

The pathophysiology differs between early- and late-onset seizures. It is important to understand these processes as treatment strategies will differ. Late seizures are often due to long-lasting structural brain changes. In contrast, early seizures often develop from electrophysiological abnormalities caused by cellular hypoxia and ischemia due to decreased blood supply poststroke and during reperfusion therapy. The current pathological mechanism of early PSE, Figure 2(a), includes electrolyte imbalance, acid-base disturbances, breakdown of the phospholipid bilayer, the release of oxidative damage from the release of free fatty acids, and increased release of the excitatory neurotransmitter glutamate [1]. The pathogenesis of late seizures is due to the instability of the blood-brain barrier (Figure 2(b)). Multiple mechanisms for the cause of late seizure include gliotic scarring with changes in membrane properties, extravasation of albumin causing the activating astrocytes and microglial cells, changes in astrocytic enzymes, and cerebral irritation from hemosiderin deposits, and thrombin stimulation of protease-activated receptor 1 [3]. Although much is still unknown about the pathophysiology of PSE, the purpose of this portion of the review article is to highlight the recent discoveries that further our understanding of early- and late-onset seizures of PSE.  Figure 3 reveals recent discoveries in the pathogenesis of PSE, including elevated interleukin-6 (IL-6) plasma concentration, immunological markers (epinephrine and high-mobility group protein B1), and low serum neuropeptide Y levels, elevated theta oscillations within the hippocampus, and cerebral small vessel disease.

#### 2.3.1. Interleukin-6 (IL-6)

Although the literature has yet to conclude whether IL-6 is associated with the specific timing of PSE, its proinflammatory role in cerebral ischemia suggests its ability to be a marker for the development of seizures. It is assumed that central and peripheral inflammation secondary to early or late stroke damages the integrity of the blood-brain barrier [1, 36]. Chronic inflammation also impairs neuronal plasticity by affecting transcription mechanisms that result in aberrant and epileptogenic circuits. Multiple inflammatory cytokines such as IL-6 and IL-1*β* have been detected in human epileptogenic tissue and cerebrospinal fluid and have been involved experimentally in the initiation and propagation of seizures [37].

IL-6 is predominantly secreted from glial cells and generates a favorable environment for the development of epilepsy by decreasing hippocampal neurogenesis and long-term potentiation (LTP), increasing gliosis and increasing the blood-brain barrier permeability [38]. Serum IL-6 levels are elevated significantly after stroke, and its chronic elevation and upregulation may contribute to the onset of epilepsy. In this study which consisted of 209 patients diagnosed with poststroke seizure after acute ischemic stroke, the average level of IL-6 mRNA was 4.87 ± 1.91, where the expression level was higher than the case group than the control group. IL-6 level was independently correlated with seizure recurrence after adjusting NIHSS scores and lesion size. Moreover, IL-6 was assessed in predicting seizure recurrence with a sensitivity of 68.57% and specificity of 75%, suggesting its applicability as a biomarker for seizure recurrence [38, 39].

#### 2.3.2. Epinephrine and High-Mobility Group Protein B1

Several inflammatory markers are elevated granulocytes and monocytes, epinephrine, high-mobility group protein B1 (HMGB-1), and CD11b implicated in developing stroke or seizures. Within the blood sample in stroke patients, serum markers of epinephrine and HMGB-1 were elevated compared to the control patients [40, 41]. Elevated epinephrine is associated with lymphopenia, subsequent infections, and an increased 3-month mortality rate (Figure 3). On the other hand, HMGB-1 is a proinflammatory death-associated molecular pattern (DAMP) that can be passively released during brain cell death or actively secreted as an alarmin from immune cells as a mediator of neuroinflammation (Figure 3). However, in patients with epilepsy not preceded by stroke, only norepinephrine was elevated, suggesting different immune alterations in patients who develop only stroke and patients who develop epilepsy either alone or subsequent to stroke [41].

In the analysis of the blood sample from stroke and seizure patients, there was an upregulation of CD32 and downregulated HLA-DR on granulocytes and monocytes (Figure 3). The downregulation of HLA-DR affects peripheral immune function by acting as an MHC class II surface receptor responsible for antigen presentation to the immune system. CD32 alteration is important in the immunological pathogenesis of stroke and epilepsy by binding to the FcyII receptor that complexes with IgG, enhancing phagocytosis via opsonization essential for bacterial infections. Further studies should identify if the immune markers such as HMGB-1 will remain elevated in poststroke epilepsy as it is elevated after a stroke episode but no alteration in the patients only experiencing epilepsy [41].

#### 2.3.3. Neuropeptide Y

Neuropeptide Y (NPY) is a 36 amino acid neuropeptide that is expressed in the central and peripheral nervous systems. It regulates homeostatic processes such as mood, appetite, angiogenesis, cerebral vasospasm, hypothalamic-pituitary axis, and sympathetic and parasympathetic activation. NPY functions as an antiepileptic chemical messenger that inhibits neuronal excitability and seizures [42]. The loss of NPY-positive cells in the hippocampus increases the severity of seizure activity in animals with posttraumatic epilepsy. Moreover, NPY is decreased in patients with acute ischemic stroke, where NPY polymorphism is correlated with ischemic stroke. Low serum NPY levels are directly proportional to the increased risk of post-ischemic-stroke epilepsy (PISE). For every 5 ng/mL increment of serum NPY, there was a 62% decrease in the incidence of PISE. By dividing groups with a 90 ng/mL serum NPY cutoff, the proportion of patients in the low NPY group (<90 ng/mL) was associated with a higher rate of PISE [43].

The incidence of PISE decreased from 90% to 60.6% to 3.5% when their NPY serum concentrations were <85 ng/mL, 85-105 ng/mL, and >105 ng/mL, respectively. In patients with abnormal video electroencephalogram (VEEG), there was a relative decrease in serum NPY levels. In conclusion, high serum NPY was favorable in treating PISE as it functions as a neuroprotectant, inhibits inflammatory molecules, contains antioxidative properties, and inhibits Ca^2+^ influx into the neuronal cell [43].

#### 2.3.4. Blood-Brain Barrier Dysfunction Increases Theta Oscillations

Blood-brain barrier dysfunction (BBBd) is a significant risk factor for the development of epilepsy. Poststroke epileptogenesis secondary to hippocampal BBBd is associated with increased theta oscillations (3-8 Hertz). CA1 pyramidal cells (PCs) have intrinsic resonant properties that allow them to respond to theta frequencies with higher voltages. While all PCs show resonance activity at hyperpolarized potential, only a subsection of PCs reveals resonance at a near-threshold potential, which is known as the perithreshold potential. PCs that have perithreshold theta resonance also possess activated muscarine-sensitive K^+^ current (I_M_) and persistent Na^+^ current (I_NaP_), which increases their action potential (AP) firing at theta frequencies by decreasing their threshold potential [44].

Moreover, in rat models that have poststroke epilepsy secondary to prothrombotic stroke induction, there is an increase in the proportion and excitability of resonant hippocampal PCs at perithreshold potential and decreased proportion and excitability of nonresonant neurons. This event facilitates a larger percentage of AP firing at the theta range, as these rats require less depolarization to reach supra- and perithreshold potentials. However, resonant PC neurons had preserved their intrinsic properties at subthreshold potentials as BBBd models had no major changes in cell morphology that could enhance their AP firing in the theta range. Vera and Lippmann results suggest that the increased AP firing from the PCs at the theta range will communicate their frequency downstream to other postsynaptic neurons to strengthen the excitable network activity and promote epilepsy-related cognitive dysfunctions. In simplicity, a greater distribution of highly excitable resonant neurons within the hippocampus is correlated to increased theta oscillations that underlie the pathogenesis of seizures in poststroke epileptic rat models [44].

#### 2.3.5. Cerebral Small Vessel Disease

Hypertension-associated cerebral small vessel disease (CSVD) increases the risk for temporal lobe epilepsy. This idea is supported by 16-week-old spontaneous hypertensive rats (SHR), a model for systemic hypertension, developing a greater likelihood of developing temporal lobe seizures after amygdala kindling. The mechanism may be due to the upregulation of angiotensin 1 and 2 receptors within the hippocampus, as early treatment with enalapril prevents the development of CSVD and decreases the inclination for the SHR to develop amygdala-kindled seizures [45]. It is thought that angiotensin peptides may interact with neurotransmitter systems such as gamma-aminobutyric acid and adenosine to facilitate the pathophysiology of small vessel disease, as earlier studies have shown the effect of losartan effect in delaying the onset of kainate-induced seizures in SHRs [46].

Furthermore, this evidence may help further substantiate uncontrolled hypertension's role in epilepsy-associated leukoaraiosis. Leukoaraiosis are abnormal white matter rarefactions that are thought to be due to small vessel disease causing occult cortical microinfarcts and are often seen either as hypodensities on CT and T1 MRI or hyperintensities on T2 and FLAIR MRI in the periventricular areas or deep white matter [46, 47]. Other studies suggest that the hypertension-associated white matter lesions may involve U-fibers, increasing susceptibility for seizures [47]. Patients with leukoaraiosis and SHR both have a predilection for temporal lobe epilepsy, which increases leukaraiosis-related epilepsy loosely with hypertensive-associated cerebral small vessel disease.

It is unclear whether leukoaraiosis is a mere incidental MRI finding or plays a role in epileptogenesis, as only a casual association has been made. This is because leukoaraiosis is a common finding the advanced adulthood, and temporal lobe epilepsy is the most frequently focal epilepsy at all ages [46, 48]. Currently, evidence of risk factors for epilepsy-associated leukaraiosis is severely lacking. However, uncontrolled hypertension may be one of the strongest associations with cerebral small vessel disease epilepsy [46, 47].

### 2.4. Pharmacological Therapies in the Treatment of PSE (Current and Emerging)

#### 2.4.1. Antiepileptic Drugs

*(1) Primary vs. Secondary Prophylaxis*. The current literature does not support the prophylactic use of antiepileptic drugs (AEDs) to prevent early- and late-onset seizures following a stroke, as they have not been found to reduce the risk of occurrence [49]. However, once a diagnosis of PSE is established, AEDs help establish seizure control with secondary prophylaxis. Clinical trials have shown that stroke-related epilepsy responds better to AED treatment than other forms of focal epilepsy [29].

In general, most physicians choose to prescribe AEDs as primary seizure control, but there is a lack of consensus on the most effective drug to prescribe due to multiple drug interactions and differences in mechanisms of action. For this reason, AED therapy must be individually tailored to each patient's comorbidities and the drugs used for their comorbidities. A Taiwanese nationwide study concluded that second generation AEDs were more effective at achieving seizure control when compared to first generation drugs. In general, first generation AEDs such as phenytoin and carbamazepine (CBZ) have a higher incidence of drug-drug interactions and a less favorable side effect profile when compared to second generation drugs such as levetiracetam (LEV) and lamotrigine (LTG) [50].

*(2) First Generation vs. Second Generation AED Treatment*. First generation AEDs are drugs first synthesized between 1912-1972 and include carbamazepine, ethosuximide, phenobarbital, phenytoin, primidone, and valproic acid (Tables 2 and 3). Many of these drugs work by modulating sodium or calcium channel activity within the central nervous system (CNS) and are metabolized by the CYP-P450 system. Because of the enzyme-inducing or inhibiting metabolism of many first generations AEDs, there are significant drug-drug interactions to consider when prescribing. There are significant reductions in plasma levels of nimodipine, a drug used to treat subarachnoid hemorrhage when administered with phenytoin, and 50% higher concentrations of nimodipine with the administration of valproic acid, which highlights the unique difficulties in treating PSE patients with first generation AEDs. First generation AEDs also interfere with statin and oral anticoagulant (OAC) metabolism. This complicates care as many stroke patients are started on these medications to prevent further cardiovascular events. Additionally, complications are generally more frequent with 1st generation drugs. Phenytoin was associated with more febrile days and worse functional outcomes at 3-month follow-up in PSE patients with ischemic stroke compared to control populations who did not receive phenytoin [29].

While first generation drugs have dominated seizure control in the past, there is now a trend towards adopting newer secondgeneration AEDs for their favorable side effect profile and decreased incidence of drug-drug interactions (Table 2). 72% of patients receiving 2nd generation AEDs maintained their original treatment regime as compared to only 36.5% of patients in the 1st generation group. This confirms that the newer drugs have much more tolerable adverse and fewer side effects. Additionally, focal epileptic patients were better able to tolerate 2nd generation drugs LEV and LTG when compared to the 1st generation drug CBZ [50–52].

Second generation AEDs also seem to be more effective at controlling seizures. 69.6% of patients in the 2nd generation AED group remained seizure-free at one-year follow-up as compared to only 38.3% in the 1st generation treatment group (Tables 2 and 3). However, there is still no consensus on the efficacy of this newer generation AEDs due to the surprising lack of large-scale studies. LEV and LTG had superior tolerability compared to CBZ, but no differences in seizure freedom were noted. It should be noted that these results come from studies with relatively small sample sizes [50–52].

*(3) Combinational AED Therapy*. A novel approach to treating and managing poststroke epilepsy is the coadministration of sodium valproate (VAL) with lamotrigine rather than using sodium valproate alone. Sodium valproate's mechanism of action is thought to increase levels of gamma-aminobutyric acid (GABA) via inhibition of GABA transaminase and succinate semialdehyde dehydrogenase, which are enzymes involved in the catabolism of GABA. Whereas lamotrigine, a new triazine antiepileptic, exerts its effect by limiting the release of glutamate via antagonizing the voltage-dependent sodium channels. The total effect rate of the study group who received combination therapy was higher than that of the control group who received VAL alone at 89.47% vs. 75.36% [53, 54].

Furthermore, patients that were coadministered of sodium valproate with lamotrigine showed less epileptic discharge and a number of involved leads shown via electroencephalogram (EEG) than patients on sodium valproate alone. The experimental group revealed a decrease in inflammatory markers: high-mobility group protein B1, matrix metalloproteinase-9, and interleukin-6, which have been implicated with poststroke epilepsy. After 6 and 12 months, quality of life scores revealed that the patients in the experimental group scored higher than those in the control group. Along with the neuronal benefits of coadministration of sodium valproate with lamotrigine, there was no significant difference in the adverse reactions when compared to the treatment of sodium valproate alone [53].

*(4) Pharmacoresistance to AED Therapy*. A study showed that out of 159 patients with PSE, 18.2% were pharmacoresistant, meaning treatment wanned in efficacy. Of the patients with ischemic poststroke epilepsy and hemorrhagic poststroke epilepsy, the rate of drug resistance was 12.8% and 28.1%, respectively. Their results revealed that patients had a higher likelihood of antiseizure medication resistance or refractoriness if they had: younger age at stroke onset, a history of intracerebral hemorrhage, severe stroke, status epilepticus at epilepsy onset, and focal to bilateral tonic-clonic seizures. They suggest that pharmacoresistance to the current gold standard antiepileptics is due to changes in voltage-gated ion channels and neurotransmitter receptors, increased production of efflux transporters, specifically ATP-binding cassette within the blood-brain barrier caused by inflammatory responses after a cerebrovascular accident. Other theories that may lead to pharmacoresistance include pathological synaptogenesis and maladaptive organization of the neuronal network that prevents antiseizure medications from targeting the overstimulated neurons. More specifically, the study indicates that old age is a protective factor to pharmacoresistance due to the decreasing neuronal plasticity with age, which limits the epileptogenicity, augmenting the response to treatment in older patients [55].

#### 2.4.2. Statins

*(1) Poststroke Epilepsy Prophylaxis*. As mentioned above, AEDs are not recommended for primary prophylaxis of PSE due to their inability to prevent neuronal changes in the acute and late phases. Interestingly, statins have been shown to be efficacious in preventing the development of PSE within specific conditions. Chiefly, these drugs have been shown to be most effective when given in the acute phase after a stroke and at higher doses than standard cholesterol-controlling doses. In a recent rat study, atorvastatin and simvastatin at a dose of 10 mg/kg/day were shown to reduce seizure incidence and suppress reactive gliosis, which is a key factor in the pathogenesis of PSE. This further supports the theory that statins could be used as a prophylactic drug to prevent PSE [56]. Stroke patients who were given statins in the acute phase showed a significantly lower incidence of ES and PSE. The same benefit was not seen in patients on prestroke statin therapy [49]. Early PSS is linked as a major risk factor for developing PSE. So, by implementing statins in the acute phase poststroke protocol, physicians may be able to prevent the progression to PSE.

According to a recent 2021 study, the antiepileptogenic effects of statins were dose-dependent. 1152 ischemic stroke patients were given either a standard dose of 20 mg atorvastatin or 10 mg rosuvastatin vs. double doses of each. Researchers followed up after 18 months to evaluate PSE incidence. They found that patients receiving the double-dose statins had a significantly reduced incidence of PSE at 0.41% in the double-dose group compared to control groups with 2.54% incidence suggesting a dose-dependent mechanism for statin's antiepileptogenic effects. The mechanism for the antiepileptogenic effects of statins is hypothesized to be involved in the maintenance of BBB integrity. Specifically, reducing IgG exosmosis, increasing the expression of endothelial nitric oxide synthase mRNA, and reducing P53, BAX, and the caspase-3 expression. Additionally, statins regulate biochemical pathways, including glycogen synthase kinase 3, which reduces mossy fibers sprouting. Statins are also thought to inhibit astrocytes' proliferation, thereby reducing the overall incidence of PSE [57].

Contrary to the findings of most studies, one study found no difference in the incidence of poststroke epilepsy among statin users (13.3/1000) and nonstatin (15.7/1000) users when conducting a retrospective cohort study of about 19000 Taiwanese stroke patients. One possible explanation is that this study only analyzed seizures occurring after discharge from the hospital, leading to an underestimation of the effects of statins. Furthermore, the study did not analyze statin doses which is a significant factor in determining the efficacy of statin treatment for PSE/ES. Finally, no drug interactions were analyzed in this study, potentially allowing patients on multiple drugs to experience an increase in the metabolism of statins and, therefore, lower efficacy due to decreased plasma concentrations [58].

**Figure 4:**
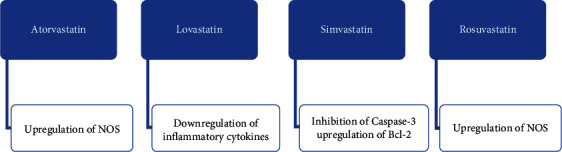
Shows the differing mechanisms of action by which various statins exude their potential antiepileptogenic effects. Atorvastatin and rosuvastatin upregulate nitric oxide synthase (NOS), while lovastatin decreases inflammatory cytokines. Simvastatin inhibits Caspase-3 and upregulates Bcl-2, thus inhibiting neuronal apoptosis.

*(2) Statin Pharmacokinetics*. Not all statins are created equal, as they vary in pharmacokinetics and metabolism. Mice models have demonstrated that statins, particularly highly hydrophobic statins, have differing MOA on neurons (Table 3). Atorvastatin decreases seizure incidence via upregulating nitric oxide synthase (NOS) activity (Figure 4). Simvastatin was shown to inhibit caspase-3 expression and induce Blc-2 expression, preventing neuronal apoptosis. Lovastatin was shown to decrease inflammatory molecules, thus reducing neural cell death.

Hydrophilic statins have also been shown to reduce epilepsy occurrence in mice models. For example, rosuvastatin was shown to maintain BBB integrity and increase NOS expression. This finding suggests that statins' hydrophobic/hydrophilic properties are not useful in determining their respective antiepileptogenic efficacy. More studies need to be conducted to elucidate the most beneficial choice of statin therapy for PSE prevention [58, 59].

*(3) Synergistic Effect with Aspirin*. Postischemic stroke patients are often put on anticoagulant drugs and statins to reduce mortality associated with the thrombotic complications of epilepsy. Doing so has been shown to alter plasma concentrations of AEDs by increasing the bioavailable concentrations. Statin use, both with and without aspirin, reduced the number of seizures in patients suffering from PSE (Table 3). Atorvastatin+aspirin combo was given to 202 PSE patients taking levetiracetam (LEV) for seizure control. This combination was associated with significantly fewer epileptic events (6.2 events at 12-month follow-up) vs. atorvastatin alone+LEV (8.6 events at 12-month follow-up) and aspirin alone+LEV (10.6 events). Using these results, researchers noted that aspirin given with atorvastatin worked synergistically to reduce seizures significantly. Aspirin+LEV was no better than LEV alone, suggesting a synergistic effect of aspirin and statin coadministration. The relationship between aspirin and epilepsy remains unclear. Mouse models suggest that aspirin's mode of action is via promoting hippocampal nerve regeneration in addition to the COX-2-PGE2 anti-inflammatory pathway [60, 61].

#### 2.4.3. Emerging Therapies

*(1) Neuropeptide Y (NPY)*. Patients who are unresponsive to traditional treatments have alternative options. Surgical resection is commonly performed in epileptic patients where drug-resistant seizures originate within the temporal lobe. However, conventional antiepileptic drugs are ineffective in treating epilepsy; administration of exogenous NPY may provide an alternative therapy by inhibiting seizure activity before more invasive neurosurgery is indicated. Neuropeptide Y (NPY) inhibits seizure-like activity on resected hippocampal tissues in patients treated for drug-resistant temporal lobe epilepsy. Initially, the resected hippocampal tissues revealed hippocampal sclerosis with neuronal loss in the CA1 pyramidal layer, neuronal cell atrophy of the dentate gyrus, and thinning of the granule cell layer [42].

The administration of NPY increases the interspike interval, decreases the regular paroxysmal depolarizing shifts (PDS), and decreases the total number of action potentials in the granule cells in the hippocampus by 60%. The stimulation of the Y2 receptors by NPY decreases glutamate release in the dentate gyrus by negative regulation of voltage-gated calcium channels. Administration of Y2 receptor antagonist BIIE0246 with NPY reveals no difference in the interspike interval, number of PDS events, and number of AP compared to baseline control. Thus, overexpression of NPY and Y2 receptors within the hippocampus may halt and reverse the frequency of status epilepticus. Exogenous NPY therapy may be an emerging gene therapy that may help reduce the epileptic activity after a cerebrovascular accident (Table 3) [42].

## 3. Conclusion

The significant risk factors for PSE include age less than 65 years, stroke severity measured by the National Institutes of Health Stroke Scale (NIHSS), cortical involvement, and genetic factors such as TRPM6 polymorphism. The IL-6, epinephrine, high-mobility group protein B1, neuropeptide Y, theta oscillations in the hippocampal neurons, and cerebral small vessel disease have been recently implicated in the pathogenesis of PSE. The use of statins and aspirin with AED as prophylaxis for PSE are accruing clinical interest. Coadministration of valproate and lamotrigine improves treatment outcomes in PSE. Moreover, second generation AED is more efficacious than that of first generation AED in treating PSE.

## 4. Limitations

The limitation of this literature review is that its primary focus is understanding the recently discovered risk factors, pathophysiology, and recent treatments of PSE. As a result, there may be only limited observational studies that result in finite supported data. Further clinical research is needed to support these case-control and cohort studies. Additionally, promising results using Neuropeptide Y require additional clinical studies to determine its safety and efficacy in humans.

## Figures and Tables

**Table 1:** Risk factors for post-stroke epilepsies.

Risk factor	Study	Epilepsy secondary to type of stroke	Participants	PSE incidence	*p*
Male sex	Sarfo et al. [16]	Ischemic and hemorrhagic	1101 Ghanaian stroke survivors	OR: 1.94	0.0008
HTN (BP > 130/80 mmHg)	Sarfo et al. [16]	Ischemic and hemorrhagic	1101 Ghanaian stroke survivors	OR: 2.26	0.08
Hemorrhagic transformation	Brondani et al., [23]	Ischemic	153 stroke patients	OR: 3.55	0.033
mRS >2	Brondani et al., [23]	Ischemic	153 stroke patients	OR: 5.82	0.013
NIHSS score (a). >4 (b). >11	Abraira et al. [25]	Ischemic and hemorrhagic with early-onset PSSE Ischemic	50 patients 4229 patients	HR: 15.757 HR: 2.33	0.019 <0.001
Cortical involvement	Ferreira-Atuesta et al. [14] Sarfo et al. [16]	Ischemic Ischemic and hemorrhagic	4229 patients 1101 Ghanaian stroke patients	HR: 2.07 HR: 1.79	<0.001 0.02
Large artery atherosclerosis	Ferreira-Atuesta et al. [14]	Ischemic	4229 patients	HR: 1.51	0.004
Acute symptomatic seizure	Ferreira-Atuesta et al. [14]	Ischemic	4229 patients	HR: 4.18	<0.001
PSSE duration >16 hours	Abraira et al. [25]	Ischemic and hemorrhagic with early-onset PSSE	50 patients	HR: 7.483	0.023
TRPM6 polymorphism	Fu et al. [6]	Ischemic	798 Chinese patients (378 PSE, 420 controls)	OR: 1.365	0.003
IL-6 mRNA expression grade 4	Jia et al. [38]	Ischemic	209 patients	HR: 13.893	<0.001
Parietal lobe involvement	Takase et al. [17]	Ischemic and hemorrhagic	93 patients	OR: 4.95	0.023
Personal history of coronary disease	Redfors et al. [5]	Ischemic	1066 patients	HR: 2.22	0.006
Aortic dissection	Redfors et al. [5]	Ischemic	1066 patients	HR: 2.65	0.002
Anterior circulation infarct	Redfors et al. [5]	Ischemic	1066 patients	HR: 26.64	<0.001
Early seizures	Redfors et al. [5]	Ischemic	1066 patients	HR: 4.57	<0.001

^∗^PSE: poststroke epilepsy; OR: odds ratio; HR: hazard ratio; HTN: hypertension; BP: blood pressure; mRS: modified Rankin scale; NIHSS: national institutes of health stroke scale; PSSE: poststroke status epilepticus; and IL-6: interleukin-6.

**Table 2:** First vs. second generation AED.

Author	Study design	Population	Interventions	Follow up	Seizure freedom	Tolerability/retention	Mortality
Tanaka et al. [50]	Prospective observational cohort study	372 Japanese patients	36 1^st^ gen 286 2^nd^ gen 50 mixed	371 days (median)	38.3% 1^st^ gen 69.6% 2^nd^ gen	36.5% 1^st^ gen 72% 2^nd^ gen	~
Hsu et al. [51]	Retrospective cohort study	6962 Taiwanese patients	5991 1^st^ gen 965 2^nd^ gen	Variable	~	~	4.9% 1st gen 2.4% 2^nd^ gen
Sales et al. [52]	Pooled analysis of retrospective/prospective	76 European patients	ESL	365 days	48.6% ESL	87.8% ESL	~

^∗^Eslicarbazepine: ESL, Gen: generation.

**Table 3:** Treatment, Mechanisms, Interactions, and Effects on Post-Stroke Epilepsy.

Drug	Mechanism	Interactions	Effects on PSE
Statins: maintenance of BBB integrity (various mechanisms) and inhibition of astrocyte proliferation			
Rosuvastatin	Upregulation of NOS	Synergistic effect with aspirin in the treatment of PSE	Lower incidence and risk of hospitalization in a dose-dependent manner
Lovastatin	Inhibition of inflammatory cytokines		
Simvastatin	Prevents neuronal apoptosis via inhibition of caspase-3 and upregulation of Bcl-2		
Atorvastatin	Upregulation of NOS		

1st generation AEDs			
Phenytoin	Blocking of voltage-gated Na^+^ channels	Enzyme Inducer: decreases nimodipine, oral anticoagulant, and statin concentrations	~
Carbamazepine	Blocking of voltage-gated Na^+^ channels	Enzyme Inducer: decreases nimodipine, oral anticoagulant, and statin concentrations	~
Valproic Acid	Blocking of voltage-gated Na^+^ channels	Enzyme inhibitor: increases nimodipine, oral anticoagulant, and statin concentrations	Increase of GABA activity due to inhibition of GABA transaminase and succinate dehydrogenase

2nd generation AEDs			
Levetiracetam	Binding to presynaptic vesicle protein 2A to inhibit excitatory neurotransmitter release	~	Reduced reactive gliosis and Inhibition of IL-1*β*
Lamotrigine	Blocking of voltage-gated Na^+^ channels	Synergistic effects with sodium valproate in the treatment of PSE	Limits glutamate release in conjunction with increased GABA due to sodium valproate's effects leading to more effective seizure control than LTG alone

Others			
Aspirin	Inhibition of COX-2-PGE2 pathways	Synergistic effect with statins in the treatment of PSE	Promotion of hippocampus nerve regeneration (mouse models)
NPY	Decreased glutamate release via negative regulation of calcium channels	~	Decreases the total number of action potentials between damaged neurons

^∗^NOS: nitric oxide synthase; Na^+^: sodium ion; PSE: poststroke epilepsy; IL-1*β*: interleukin-1*β*; GABA: *γ*-aminobutyric acid; LTG: lamotrigine; COX-2-PGE2: cyclooxygenase-2-prostaglandin E2.

## Data Availability

No underlying data was collected or produced in this study.
